# Development of the conceptual framework for the Eye-Drop Satisfaction Questionnaire (EDSQ^©^) in glaucoma using a qualitative study

**DOI:** 10.1186/1472-6963-7-124

**Published:** 2007-08-06

**Authors:** Jean-Philippe Nordmann, Philippe Denis, Marc Vigneux, Elyse Trudeau, Isabelle Guillemin, Gilles Berdeaux

**Affiliations:** 1 Hôpital des XV XX, Paris, France; 2 Hôpital Edouard Herriot, Lyon, France; 3 Mapi Values, Lyon, France; 4 Conservatoire national des Arts et Métiers, Paris, France; 5 Alcon France, Rueil-Malmaison, France

## Abstract

**Background:**

Compliance is a major issue in glaucoma care. It is usually poor in glaucomatous patients, and may ultimately result in an acceleration of the disease progression and a risk of blindness. Reasons for this poor compliance are complex and multifactorial, amongst which patient satisfaction can be counted. The objective of this study was to develop a questionnaire to assess patient satisfaction and compliance with eye-drop treatment.

**Methods:**

A qualitative study was carried out to develop the questionnaire. An interview guide was developed based on a literature review. Structured interviews of fifteen French and English patients with primary open-angle glaucoma or intraocular hypertension were conducted by trained interviewers of the native language of the interviewees. General concepts and subconcepts were identified from the transcripts. The questionnaire was developed using the patient verbatim, and submitted to six patients (French and English) for cognitive debriefing. Following patients' comments, items were modified and restructured, and a pilot questionnaire was designed.

**Results:**

Analysis of data from the interviews with patients and clinicians resulted in the elicitation of concepts related to patient satisfaction and compliance with glaucomatous treatment. These were further refined and used to generate a test questionnaire, which consisted of 46 items grouped into 6 domains: patient characteristics, treatment characteristics, patient-clinician relationship, patient experience with the disease and the treatment, interaction between the patient and the treatment, and patient knowledge of the disease and the treatment.

**Conclusion:**

The Eye-Drop Satisfaction Questionnaire (EDSQ) conceptual framework and items were developed simultaneously in French and in English. This questionnaire could be used to evaluate patient satisfaction and compliance with eye-drop treatment and would facilitate the identification of patients at risk of being non-compliant prior to clinical trials or innovative device tests. A psychometric study is under way to validate the questionnaire.

## Background

Primary open-angle glaucoma (POAG) is the most common form of glaucoma. It is a chronic disease characterised by a progressive alteration of the optic nerve, leading to visual impairment. Glaucoma is one of the three leading causes of visual impairment in developed countries [1,2]. The prevalence of glaucoma is about 2% in people over 40 years and increases with age [3-5]. Reported increase in office visit rates is also directly associated with ageing [6]. According to the World Health Organisation, visual impairment was responsible for 2,286,000 disability-adjusted life years in high-income countries in 2001 [7]. It has considerable impact on the economy because of the non-medical costs associated with the incapacity and dependency resulting from the disease, such as loss of income, burden on carer, paid assistance and residential adaptation [8]. Visual impairment also negatively affects the Health-Related Quality of Life (HRQoL) of patients [9,10], and constitutes a major cause of disability that affects individuals, family and society, and is associated with an increased risk of institutionalisation and death [1,11-13].

In all prospective multi-centre trials, elevated intraocular pressure (IOP) is a major risk factor of POAG, even though up to 50% of reported glaucoma cases may have "normal" or "average" IOP. Moreover, elevated IOP has been observed in patients with ocular hypertension (OHT), who may never develop glaucoma [14,15]. However, multiple randomised studies have established the efficacy of IOP reduction in preventing glaucomatous progression [16-19]. Therefore, control of IOP remains the main approach to glaucoma care, and several medical strategies have been developed to improve care: medication, laser treatment and surgery. Treatment of glaucoma or OHT with medications is a major factor in the control of the progression of glaucoma, thus contributing to the maintaining of patients' HRQoL [13,20-22]. Nevertheless, non-compliance of glaucomatous patients with the prescribed therapy is significant, and is a highly limiting factor in the management of glaucoma. Numerous barriers to medication compliance exist, such as the lifelong-nature of the treatment, situational/environmental factors (e.g. travelling), medication regimen (e.g. difficulty in self-administering eye-drop product; side effects), patient factors (e.g. forgetfulness, wrong idea of their medical condition) and provider factors (e.g. poor communication between physicians and patients) [20,23-26]. While compliance with treatment is indeed multifactorial [27], Woodside et al. have proposed and illustrated the relationship between satisfaction and behavioural intention and attitude regarding health [28]. Specifically, glaucoma patient satisfaction with his/her treatment has been shown to be a key driver of compliance [29], and could therefore be used as an endpoint to characterise patients at risk of being non-compliant. This information could help characterize the treatment features that need to be improved by identifying the determinant of satisfaction.

To this end, we proposed to develop a questionnaire on compliance and satisfaction with medications administered as eye-drops (the Eye-Drop Satisfaction Questionnaire – EDSQ-) for glaucoma or IOP treatment. Recently, the importance of the very first steps in the development of a Patient-Reported Outcomes (PRO) questionnaire has been pointed out, and includes the design of a thorough and rigorous conceptual framework following patient verbatim [30]. This manuscript presents the development of the conceptual framework based on patient and clinician interviews, and the subsequent generation of the items that constitute the EDSQ.

## Methods

### Concept development

Based on patient concerns about eye-drop treatment identified in the literature review, a project team consisting of a health psychologist, two ophthalmologists and a group of experts in the development of questionnaires first met to design a list of concepts related to patients' expectations and satisfaction with eye-drop treatment. This concept list was used for the development of the clinician and patient interview guides.

#### Clinician interviews

Five clinicians who had experience in the treatment of patients suffering from glaucoma or ocular hypertension were recruited. Interviews with the clinicians were conducted either face-to-face (France; n = 3) or by phone (United Kingdom; n = 2) by a health psychologist and a trained reviewer, and lasted 30 to 45 minutes. Clinicians received a financial incentive for their participation.

The clinician interview guide explored the following topics: clinicians' perception of the disease (s), patient management, patients' expectations regarding their management, ease of use and treatment constraint, compliance with the treatment and patient education about the disease and its treatment.

#### Patient interviews

At the time of their medical visit to their ophthalmologist, patients were asked to participate in the study. They had to be aged between 50 and 75 years; diagnosed with OHT; and be treated with one or more ophthalmic drop(s). Prior to the interview, all the patients included had to sign an informed consent form. Fifteen voluntary patients (n = 9 in France and n = 6 in the United Kingdom) were recruited, in private practices for French patients, and in outpatient clinics for English ones. One-hour interviews were conducted by a health psychologist (in France), and a trained interviewer (in England) in the native language of the interviewees and interviewers, and were tape-recorded. These took place at the same place patients were recruited, (i.e. clinic or clinical practice), or at the patients' home. Patients received a financial incentive for their participation.

The patient interview guide explored the following points: patients' perception of the disease, fear and worries linked to the disease, patients' actual experience of the treatment, ease of use and treatment constraint, efficacy and tolerability of the treatment, information and communication between clinicians and patients, compliance with the treatment.

#### Analysis of clinician and patient interviews

The tapes of the patient and clinician interviews were first transcribed. Then, the transcripts were analysed by the health psychologist following the Interpretative Phenomenological Analysis approach [31], and used to amend and complete the initial list of concepts. The concepts within this second list were categorised into new global concepts and detailed subconcepts to create a conceptual framework.

### Item generation

Based on the pooled data from French and English patient verbatim extracted from the interview transcripts, the items were simultaneously generated in French and English during a project team "item generation meeting" [32]. This process ensured that each item in the subsequent questionnaire was relevant to both English and French patients.

Following discussions between members of the project team about the concepts and the choice of appropriate response-scales and of the overall structure of the questionnaire, a first version was developed.

### Evaluation of the content validity

Patient structured interviews were performed by native speaking and trained interviewers to assess the questionnaire's clarity and ease of comprehension, cultural equivalence and relevance of the questions. Participants were different from those who took part in the concept development phase, but were recruited using the same method and criteria as those used for the recruitment of patients for the first set of interviews.

Six face-to-face interviews (three per country) were performed. Two patients were diagnosed with OHT and four with POAG. Interviews were tape-recorded. During the interviews, patients were asked questions on their understanding and the wording of the items. In addition, they were asked to review each item of the questionnaire in order to evaluate the clarity and ease of understanding on first reading and the relevance of the items. Throughout the interview, the interviewer prompted the patient to ensure they gave as much feedback as possible.

## Results and discussion

Interviews were performed between December 2005 and January 2006.

### Patients' characteristics

Socio-demographic and clinical characteristics of the patients are summarised in Table 1. Of the fifteen patients interviewed, seven were diagnosed with OHT and eight with POAG. Eight patients were male and seven patients were female. The mean age of the patients was 68 years (± 10 years); the majority of them were retired. French patients had been diagnosed 16 years ago (± 9 years) on average, and had been following a treatment for 15 years on average. All patients were taking at least one type of ophthalmic drop. One French patient was suffering from tachycardia, and one from hypertension. Amongst English patients, one was suffering from prostate cancer, one with high blood pressure, one had breast cancer and one had arthritis.

**Table 1:** Socio-demographic and clinical characteristics of the patients

Characteristics		France (n = 9)	United Kingdom (n = 6)	Total (n = 15)
Pathology	Ocular Hypertension	4	3	7
	Primary Open-Angle Glaucoma	5	3	8
Time since diagnosis (years ± SD)		15 ± 9	NA*	NA
Age	Min-Max	50–82	59–81	50–82
	Mean (years ± SD)	67 ± 12	70 ± 8	68 ± 10
Gender	Male	6	2	8
	Female	3	4	7
Living situation	Alone	1	1	2
	As a couple	7	5	12
	Married	1	0	1
Working status	Retired	6	6	12
	Currently working	3	0	3

*, not available

### Concept elicitation

Therapeutic compliance is a major factor in the treatment of glaucoma and prevention of blindness. However, compliance remains poor in this disease area; patient satisfaction has been identified as being one of the causes [28]. Based on these observations, we have developed a satisfaction questionnaire on eye-drop treatment that will help identify patients at risk of being non-compliant.

**Figure 1:**
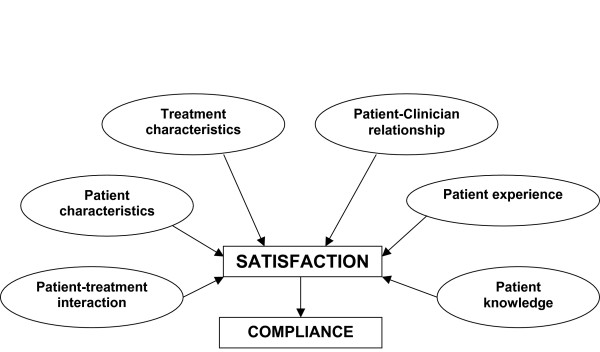
Conceptual framework for the Eye Drop Satisfaction Questionnaire.

Qualitative analysis of pooled data from English and French patients' and clinicians' interviews revealed six major concepts. Some of these had already been addressed and gathered from the preliminary literature review, but interviews also allowed the elicitation of more emotional aspects by placing the patient in the psycho-sociological context of the disease. The conceptual framework is presented in Figure 1. Concepts extracted from the clinicians' and patients' interviews are presented in the following sections, and are illustrated with selected citations from the interviews. Where French verbatim was included, this was translated into English by a bilingual English speaking native. This verbatim is annotated with an asterisk in the text.

#### Patient characteristics

According to the clinicians' interviews, "non-compliant (patients) are more often men*", a "retired 'old man' is more compliant*", and "non-compliant (patients) are more often young*". Patients reported physical difficulty in self-administering drops ("the actual drop itself can be difficult to put in"; "to put the drops in by myself would be difficult*", "Yes, perhaps for people who are unsteady; this is not my case*"), which could result in missing the eye ("quite often it falls on the eye lid and trickles away so I have to do it again"), and their discomfort in having to put things in their eyes: "I hate instruments that get close to the eyes*". Related to these remarks may be patients mentioning that the presence of somebody (spouse, husband or external help) would facilitate this step ("my mother applied the drops to my father's eyes*"). Clinicians also expressed the discomfort and difficulty of the drop instillation process ("they just seem to say that it's hard to look in the mirror and hold one eye at the same time as putting a drop in-so it's the coordination of the whole thing – very rarely someone says that the bottle itself is a problem, but that's rare"; "they find it hard to put them in themselves"; "if someone was coming in regularly to help them put the drop in they would be able to do it"), and have observed that "plenty of those people will have them put in by their wife or spouse". Factors related to travel, whether professional or personal, constituted another major reason of non-compliance with the treatment. Some patients reported difficulties in having to remember to take their bottle when they were away: "always having my bottle with me", "even in another country", "I have forgotten them when I have been away from home". Clinicians also indicated that "professional life makes the follow-up of the treatment difficult*". A few patients explained that they were compliant with their treatment because a person in their family or friends had previously had glaucoma ("My dad having had glaucoma that was badly treated. He was 80 years old and maybe that at this age, you have other priorities. I knew I had to be very careful and pay great attention to my condition*"). Finally, clinicians reported that non-compliance could be explained by "the patients' attitude first of all, and usually they are some other issues going on that make them not bother to put the drops in".

Thus, patient characteristics including socio-demographic criteria such as age and gender appear to have a direct impact on eye-drop treatment compliance. Their level of education also has an impact (data not reported). In particular, the marital status seems to be an important factor in compliance; the presence of a partner, or any external help, is a motivating factor for patients, either as a reminder or as a direct participant who would administer eye-drops. Previous glaucoma history in patients' entourage may help in increasing their compliance, as the patient could then be aware of the effects that might result from an inappropriate treatment. Physical difficulties including palsy, discomfort putting things in eyes, and blink reflex when instilling the drops are a barrier regarding the patients' compliance with eye-drop treatment. Patient compliance is also related to the time they can spend administering their drops during the day. Lastly, travel or any modifications in patient habits often result in forgetting to use their medication, and thus have a negative impact on compliance.

#### Treatment characteristics

Daily frequency of the treatment was often given by patients as having an impact on their compliance: "three (drops) in the evening and two in the morning with a ten-minute pause between the two drops", "the one (drop) during the day, I might forget it", "the one in between I might take it at dinner time and might take it at 4 o'clock". Side effects and feelings of discomfort resulting from the treatment were also reported by patients and clinicians: "It stings. I don't want to take my drops tonight", "It stings and it makes my eyes red. I'm going out tonight, I don't want to have red eyes*", "It burned when I applied it. I had puffy eyes, red eyes*".

Patients' compliance and/or satisfaction with the treatment may be influenced by the characteristics of the treatment itself, such as the frequency of daily intake, and the time of intake. The amount and intensity of the treatment side effects are major factors: the greater and more painful the side effects, the less compliant the patients. Finally, satisfaction is closely related to compliance; indeed, if the patient is satisfied with the treatment in its overall features, then he/she will be more compliant.

#### Patient-clinician relationship

Sharing information about the disease and the treatment with their clinician and checking-up on the efficiency of the treatment have been mentioned by most patients as motivating factors for compliance and/or satisfaction: "(About change in the visual field) without an ophthalmologist it is impossible to know about it on your own*", "I know because every 6 months, I have an appointment with my clinician and he measures my ocular tension. And I noticed that from 17, I stabilized at 10 for each eye and it's not changing anymore. For me, this is a good result*"; A few patients said that they would be encouraged to continue their treatment if their physicians were willing to train them how to administer their medication and gave regular check-ups. The attention paid by the clinician and positive feedback were also reported by patients as motivating factors ("I consulted a specialist who cheered me up, he told me that if the pathology is treated early enough, it couldn't go badly*"; "I was diagnosed very early. It's lucky the ophthalmologist had a good reaction, because some of them think that you are making it up*").

A good relationship, including care, training, feedback and regular check-ups with their clinician is essential to patient satisfaction, and is likely to result in improved compliance. This may be linked to the patients' difficulty in evaluating the efficacy of their treatment themselves because of the elusiveness of the disease. Only a clinician (ophthalmologist) can inform patients about a change in their medical condition (an improvement, deterioration or stabilisation). A relationship based on trust and regular check-ups of patients by their clinician is therefore necessary to motivate patients to take their treatment.

#### Patient experience

The trouble the patients have remembering to take the treatment, ("the constraint is thinking about it*"; "You get in, you fall asleep, you wake up later and can't remember if you have taken your drops") and the burden of administering drops in the eyes ("I can drive but it bothers me at night, the car lights...that's why we stopped going out in the evening*", "The only trouble is remembering to use them") were just part of the list of constraints that may result in not taking the medication. Patients expressed their doubts about the effectiveness of the treatment because of the absence of an immediate medical deterioration in their condition if they don't take their drops ("I didn't take all [my drops]) last week and feel fine"), because of the absence of a fast and noticeable improvement in their vision ("The difficulty is knowing if the treatment is working*"; "sometimes because of poor efficacy of the drops"). One patient reported his non-compliance with any medication ("I don't actually like taking medication, full stop"). Fear is very frequently reported as being a factor contributing to the non-compliance of patients. For clinicians, the association of glaucoma with blindness was the most frequent fear found in glaucomatous patients ("I think their first fear is 'am I going to go blind or not?"). For the majority of the patients, the fear of going blind was also the biggest fear linked to their medical condition ("Yes, scared of going blind, more than of being in a wheelchair*"; "I was worried at first, the fear of going blind*"). Fear of surgery was also brought up. Clinicians reported that they used these fears to incite patients to be compliant with the eye-drop treatment ("Once they have gone through that, then you can tell them about the medical treatments – we tell them that most of the time the drops work"; "Fear of surgery is a real fear; We use that to make sure they put the drops in"). Equally, having to take a treatment for life was another factor that patients indicated: "At the beginning, it makes me think, a lifelong treatment is never something pleasant*".

Thus, patient experience with eye-drop treatment may influence their compliance with it. Especially, compliance is likely to be jeopardised if the treatment is perceived as a constraint or if the patient has doubts about the effectiveness of the treatment. Furthermore, fear of the disease may also be a barrier to compliance: if the fear regarding the illness and its consequences is too high, anxiety may override the defensive mechanisms and lead to a denial of the disease that could ultimately result in renunciation of the treatment.

#### Patient knowledge

Clinicians reported that patients have no or incorrect knowledge about glaucoma and its symptoms ("A strong non-compliance factor is the non-understanding of the disease by the patient; there is no need to take treatment, I don't feel symptoms*"; "Just preconceptions they have about going blind basically and that is associated with the word glaucoma"). Patients often indicated the lack of information regarding their disease: "I have no information*", "As with a lot of treatments, in medicine, they are not very talkative about why you have to take a treatment, what it does, the inconveniences, you don't know any of these*". Patients confessed their attempts to self-diagnose their disease: "I read in a book about glaucoma symptoms about seeing a halo around the light and thought, I have that symptom, so went to see the doctor", "Oh yes because I always – they have a leaflet to tell you exactly what they are doing for you and what the side effects might be, if you have any, which was good, nothing to get upset about"), and their need to be informed (talking about information: "No, not much. I'd like to have more. But we read a lot of things. My sister is also under treatment*".

Patients' knowledge about the disease treatment also influences their compliance: patients will be satisfied if they are regularly informed about their illness and in turn will be more compliant with their treatment. To fully understand the importance of being compliant with the treatment, patients need to understand the reasons for which they have to follow a treatment, especially when the treatment has constraints. Consequently, clinicians play a major role by supplying this information, which underlines the need for a good relationship between patients and clinicians.

#### Patient-treatment interaction

Patients mentioned forgetting to take the treatment or deliberately interrupting it: "Sometimes I don't want to. I told you it happened 3, 5, 10 times maximum in 3 years... There is no reason... I think I did it consciously, so few times in 3 years; it's neutral (without consequences). ... Because I don't want to! I have children, and when they were at school, I always told them – during the school year you can take 2 mornings off no more...I do the same with [name of the product]*". Other patients said they have developed routines and organisational means to help them remember to take their treatment: "When I get up in the morning I usually have a wash and come back into the kitchen and put one drop in my right eye"; "Well, I keep the bottle on the bedside table so that usually it reminds me"; "You just get into the routine – twice a day and it's no big deal". But often, patients related these routines to a necessity, and as such defined them as an obligation: ("But I have to admit that drops every evenings, it's a constraint*"). The repetitive aspect of the treatment could also help the patient to accept the treatment, as reported by a patient: "No, it's not restrictive because one drop in each eye once a day before going to bed, it's a reflex, it's a habit and it only takes 8 seconds*". The inconvenience of the application device was raised as a problem for correctly measuring out the drops ("the bottles are not too bad. It's a mass-produced drug and with some of them, you have to be careful because there are at least 2 to 3 drops that come out*"). Its inconvenience was also noted because of the difficulties in using it for elderly people (talking about elderly people: "The bottles are very small and so, when they press them they don't have the same flexibility that we have in their fingers, and also, to have to lean back your head can result in dizziness, they may fall. That elderly people are non-compliant with these kinds of treatments doesn't surprise me*"), or because of their difficulty in administering the drops by themselves: "I was doing it myself, but watching the mirror is not very handy and I was missing my eyes. However, for some other patients, "the bottle is not too badly designed*".

Interaction between patients and treatment is another factor to consider in patients' treatment compliance. On the one hand, routines may help the patient to be compliant, but on the other hand, these same routines might be perceived as a constraint. Non-compliance might thus be a way for patients to re-appropriate their life and to cope with the anxiety and fear they associate with their disease. The convenience of the application device and its ease of use are an other major factor in satisfaction and/or compliance with the treatment.

### Item generation

Based on the conceptual framework and the verbatim of the French and English patients' pooled-responses, a pilot questionnaire was developed simultaneously in French and in English. The questionnaire contained 56 items assessing the following six concepts: a/Patient characteristics, (18 items), b/Treatment characteristics (6 items), c/Patient-clinician relationship (12 items), d/Patient treatment experience (8 items), e/Patient-treatment interaction (8 items), and f/Patient compliance (4 items). The interviewees answered using to a 5-point Likert scale ranging from "not at all" to "extremely" or from "never" to "always".

### Evaluation of the content validity

Cognitive debriefing was performed by interviewing six patients with glaucoma (n = 4) or OHT (n = 2), in France and in England. Overall, the majority of the items were well understood and accepted by patients who made few remarks regarding their difficulty in understanding and completing the questionnaire. Twelve items were deleted as they were not relevant or were redundant, and twelve questions had to be reworded or slightly modified in order to make them clearer for the patients. Following experts' comments, the order of the items was also modified in order to facilitate the completion of the questionnaire. The definition of the concepts was also refined and restructured. These concepts and the associated subconcepts are presented in Table 2.

**Table 2:** General concepts and subconcepts of the final questionnaire

**General concepts**	**Subconcepts**	**Item contents**
Patient characteristics	Gender	Patient sex
	Age	Patient age
	Marital status	Patient alone or in couple
	Level of education	Patient level of education
	Professional activities	Professional status
		Number of working hours
	Daytime availability	Free time during the day to take drops
	Travel (Professional/Personal)	Frequency of night spent away from home
		Frequency of long journeys
		Ease of prescription renewal when away
	Family environment	Self-administering or external help
		Previous experience with IOP or POAG in family or friends
	Physical difficulties	Physical difficulties such as shaking, arthritis...
	Apprehension	Discomfort
		Worries putting things in eyes
		Blink reflex
Treatment characteristics	Intake frequency	Number of times a day the drops are taken
	Time of the intake	Time of the day the treatment is taken
	Multiplicity of treatment	Number of different ophthalmic drops
	Side-effects	Presence of side-effects
Patient-clinician relationship	Visit frequency to the clinician	Number of visits per year
	Satisfaction with visit frequency	Satisfaction with the delay between two visits
	Training	Training in drop instillation
	Clinician care	Satisfaction about clinician care
	Feedback and motivation	Relation between feedback and motivation
	Follow-up and motivation	Relation between clinician follow-up and motivation
Patient experience	Treatment as a burden	Burden of the treatment
	Fear regarding the disease	Fear of the disease evolution
	Diificulty in taking drops	Satisfaction about the administration route
	Thinking constantly about the disease	Frequency of thinking about disease consequences
	Feeling about lifelong treatment	Perception as a constraint
	Confidence in the treatment	Confidence in the effectiveness of the treatment
	Forgetting the treatment	Frequency treatment is forgotten
Patient-treatment interaction	Administration route	Ease of medication use
		Convenience of the delivery system in bottle opening
		Convenience of the delivery system in drop dosing
		Convenience of the delivery system in checking quantity of drops left in bottle
		Storage of eye drops in good conditions
	Routine	Set up of routine for remembering to take drops
		Daytime intake
	Break in the treatment	Voluntary treatment break
	Self-assessment compliance	Compliance with treatment over a given period
Patient knowledge	Information received on disease and treatment	Satisfaction about the quantity of information given on the treatment
		Satisfaction about the quantity of information given on the disease
		Frequency of information given by clinician to patient about eye-pressure level
		Frequency of information given by clinician to patient about visual field

The resulting EDSQ questionnaire, available in French and English, contains 46 items, assessing 6 domains: 1/Patient characteristics (16 items, including items on age, gender, family and working status, travel, physical difficulties and apprehension), 2/Treatment characteristics (4 items, including items on frequency and time of intake, multiplicity of treatments and side-effects), 3/Patient-clinician relationship (6 items, including items on frequency of visits, satisfaction with frequency of visits, training, satisfaction with clinician care and impact of good feedback and follow-up), 4/Patient experience (7 items, including items on perceived constraints of the treatment, fear of the disease, confidence in the treatment, forgetting the treatment, difficulty in taking drops and thinking constantly about the disease), 5/Patient-treatment interaction (9 items, including items on the ease and convenience of the administration route, set up of routine, break in the treatment, and self-assessment of compliance), and 6/Patient knowledge (4 items related to the information received by the patient on the disease and the treatment). For ease and clarity, the questionnaire was divided into two distinct parts, the first part dealing with socio-demographic items, and the second with disease-related questions. Respondents answered each item using either a dichotomous scale (Yes/No) or a 5-point Likert type scale ranging from "not at all" to "extremely" or from "never" to "always".

## Conclusion

A conceptual framework and items were generated according to the Guidance for Industry document published by the Food and Drug Administration [30], and resulted in the development of the EDSQ. To our knowledge, there is no article describing the very first steps of the development of a questionnaire on glaucoma eye-drop treatment compliance and satisfaction. In this manuscript, the elaboration of a conceptual framework and the item generation based on a qualitative study of interviews with glaucomatous patients are presented. The concepts and subconcepts established herein support previous quantitative studies that have provided evidence that patient satisfaction may be related to compliance, perceived effectiveness of treatment, side effects, ease and convenience of use, acceptance of illness, and knowledge of glaucoma [29,33,34]. Two limitations to the present work should be noted; one may reside in the absence of saturation as interview methodology [30,31,35]. However, the conceptual framework that was elaborated from the preliminary literature review and subsequent patients' and clinicians' interviews appeared consistent and solid; this framework was further confirmed with cognitive debriefing. One should add that recent work has found that saturation occurs within the first twelve interviews [36]. The other limit might consist in the bias that could have been introduced when translating French verbatim into English before analysis. This may have resulted in the loss or misinterpretation of some information, although such issues were not highlighted during cognitive debriefing. Further psychometric validation is required to confirm the items.

This questionnaire will be useful to evaluate satisfaction and compliance for patients receiving eye-drop treatment for ocular hypertension and glaucoma, thus facilitating the identification of patients at risk of being non-compliant, and ultimately help to select the best patient candidates for eye-drop treatment. In the current economic climate, such a tool will render decision-making easier for pharmaceutical industries that will want to test innovative devices and newly developed treatments. A psychometric validation of the EDSQ questionnaire is under way to examine its psychometric properties; in addition, the questionnaire is being currently translated and linguistically validated in Dutch, Spanish and Italian, which will widen its use to international studies.

## Competing interests

The author(s) declare that they have no competing interests.

## Authors' contributions

JPN and PD participated in patients' interviews and manuscript reviewing; MV carried out patients' interviews and performed the verbatim analyses; ET participated in the questionnaire conception and the study coordination, and the reviewing of the manuscript; IG wrote the manuscript and gave input to the data analysis; GB conceived the study and participated in its design, as well as in the analysis and the development of the questionnaire, and in the manuscript reviewing.

## Pre-publication history

The pre-publication history for this paper can be accessed here:



## References

[B1] Berdeaux G, Brézin A, Fagnani F, Lafuma A, Mesbah M (2007). Self-reported visual impairment and mortality: a French nationwide perspective. Ophthalmic Epidemiol.

[B2] Goldstein H, Freemann JS (1990). Magnitude and causes of blindness: Sources and limitations of data. Clinical ophthalmology Volume 2.

[B3] Brézin AP, Lafuma A, Fagnani F, Mesbah M, Berdeaux G (2005). Prevalence and burden of self-reported blindness, low vision, and visual impairment in the French community: a nationwide survey. Arch Ophthalmol.

[B4] Friedman DS, Wolfs RC, O'Colmain BJ, Klein BE, Taylor HR, West S, Leske MC, Mitchell P, Congdon N, Kempen J, Eye Diseases Prevalence Research Group (2004). Prevalence of Open-Angle Glaucoma among adults in the United States. Arch Ophthalmol.

[B5] Tuck MW, Crick RP (1998). The age distribution of primary open angle glaucoma. Ophthalmic Epidemiol.

[B6] Schappert SM (1995). Office visits for glaucoma: United States, 1991–1992. Adv Data.

[B7] Mathers C, Lopez A, Stein C, Ma Fat D, Rao C, Inoue M, Shibuya K, Tomijima N, Bernard C, Xu H Deaths and disease burden by cause: global burden of disease estimates for 2001 by world bank country groups. http://www.dcp2.org/file/33/wp18.pdf.

[B8] Lafuma A, Brézin A, Lopatriello S, Hieke K, Hutchinson J, Mimaud V, Berdeaux G (2006). Evaluation of non-medical costs associated with visual impairment in four European countries: France, Italy, Germany and the United Kingdom. Pharmacoeconomics.

[B9] Odberg T, Jakobsen JE, Hultgren SJ, Halseide R (2001). The impact of glaucoma on the quality of life of patients in Norway. II. Patient response correlated to objective data. Acta Ophthalmol Scand.

[B10] Spaeth G, Walt J, Keener J (2006). Evaluation of quality of life for patients with glaucoma. Am J Ophthalmol.

[B11] Brézin A, Lafuma A, Fagnani F, Mesbah M, Berdeaux G (2004). Blindness, low vision and other handicaps as risk factors attached to institutional residence. Br J Ophthalmol.

[B12] Lafuma A, Brézin A, Fagnani F, Mimaud V, Mesbah M, Berdeaux G (2006). Nonmedical economic consequences attributable to visual impairment: A nation-wide approach in France. Eur J Health Econ.

[B13] Nordmann JP, Viala M, Sullivan K, Arnoult B, Berdeaux G (2004). Psychometric validation of the National Eye Institute Visual Function Questionnaire – 25 (NEI VFQ-25) French version: in a population of patients treated for ocular hypertension and glaucoma. Pharmacoeconomics.

[B14] Armaly MF (1969). Ocular pressure and visual fields. A ten-year follow-up study. Arch Ophthalmol.

[B15] Kitazawa Y, Horie T, Aoki S, Suzuki M, Nishioka K (1977). Untreated ocular hypertension. A long-term prospective study. Arch Ophthalmol.

[B16] Collaborative Normal-Tension Glaucoma Study Group (1998). Comparaison of glaucomatous progression between untreated patients with normal-tension glaucoma and patients with therapeutically reduced intraocular pressures. Am J Ophthalmol.

[B17] Heijl A, Leske MC, Bengtsson B, Hyman L, Bengtsson B, Hussein M, Early Manifest Glaucoma Trial Group (2002). Reduction of intraocular pressure and glaucoma progression: results from the Early Manifest Glaucoma Trial. Arch Ophthalmol.

[B18] Kass MA, Heuer DK, Higginbotham EJ, Johnson CA, Keltner JL, Miller JP, Parrish RK, Wilson MR, Gordon MO (2002). The Ocular Hypertension Treatment Study: A randomized trial determines that topical ocular hypotensive medication delays or prevents the onset of primary open-angle glaucoma. Arch Ophthalmol.

[B19] The AGIS investigators (2000). The advanced glaucoma intervention study (AGIS): 7. The relationship between control of intra-ocular pressure and visual field deterioration. Am J Ophthalmol.

[B20] Kosoko O, Quigley HA, Vitale S, Enger C, Kerrigan L, Tielsch JM (1998). Risk factors for noncompliance with glaucoma follow-up visits in a residents' eye clinic. Ophthalmology.

[B21] Stewart WC, Konstas AG, Pfeiffer N (2004). Patient and ophthalmologist attitudes concerning compliance and dosing in glaucoma treatment. J Ocul Pharmacol Ther.

[B22] The European Glaucoma Society Terminology and guidelines for glaucoma.

[B23] Denis P, Lafuma A, Berdeaux G (2004). Medical outcomes of glaucoma therapy from a nationwide representative survey. Clin Drug Investig.

[B24] Nordmann JP, Auzanneau N, Ricard S, Berdeaux G (2003). Vision related quality of life and topical glaucoma treatment side effects. Health Qual Life Outcomes.

[B25] Sleath B, Robin AL, Covert D, Byrd JE, Tudor G, Svarstad B (2006). Patient-reported behaviour and problems in using glaucoma medications. Ophthalmology.

[B26] Tsai JC, McClure CA, Ramos SE, Schlundt DG, Pichert JW (2003). Compliance barriers in glaucoma: a systematic classification. J Glaucoma.

[B27] Hays RD, Kravitz RL, Mazel RM, Sherbourne CD, DiMatteo MR, Rogers WH, Greenfield S (1994). The impact of patient adherence on health outcomes for patients with chronic disease in the medical outcomes study. J Behav Med.

[B28] Woodside AG, Frey LL, Day RT (1989). Linking service quality, customer satisfaction, and behavioral intention. J Health Care Mark.

[B29] Day DG, Sharpe ED, Atkinson MJ, Stewart JA, Stewart WC (2006). The clinical validity of the treatment satisfaction survey for intraocular pressure in ocular hypertensive and glaucoma patients. Eye.

[B30] Patrick DL, Burke LB, Powers JH, Scott JA, Rock EP, Dawisha S, O'Neill R, Kennedy D Patient-Reported Outcomes to support medical product labelling claims.

[B31] Charmaz K, Smith JA, Harré R, Van Langenhove L (1995). The Grounded Theory. Rethinking methods in psychology.

[B32] Kubin M, Trudeau E, Gondek K, Seignobos E, Fugl-Meyer AR (2004). Early Conceptual and linguistic development of a patient and partner Treatment Satisfaction Scale (TSS) for erectile dysfunction. Eur Urol.

[B33] Atkinson MJ, Sinha A, Hass SL, Colman SS, Kumar RN, Brod M, Rowland CR (2004). Validation of a general measure of treatment satisfaction, the Treatment Satisfaction Questionnaire for Medication (TSQM), using a national panel study of chronic disease. Health Qual Life Outcomes.

[B34] Atkinson MJ, Stewart WC, Fain JM, Stewart JA, Dhawan R, Mozaffari E, Lohs J (2003). A new measure of patient satisfaction with ocular hypotensive medications: The Treatment Satisfaction Survey for Intraocular Pressure (TSS-IOP). Health Qual Life Outcomes.

[B35] Morse JM (1995). The significance of saturation. Qual Health Res.

[B36] Guest G, Bunce A, Johnson L (2006). How many interviews are enough? An experiment with data saturation and variability. Field Methods.

